# Supplementary figures and images for: Unilateral step training can drive faster learning of novel gait patterns
Source: Sci Rep. 2020 Oct 29;10:18628. doi: 10.1038/s41598-020-75839-3 (PMC7596053; doi:10.1038/s41598-020-75839-3)

A

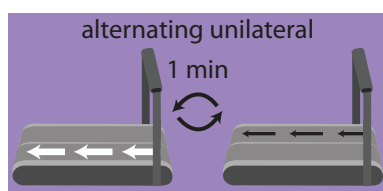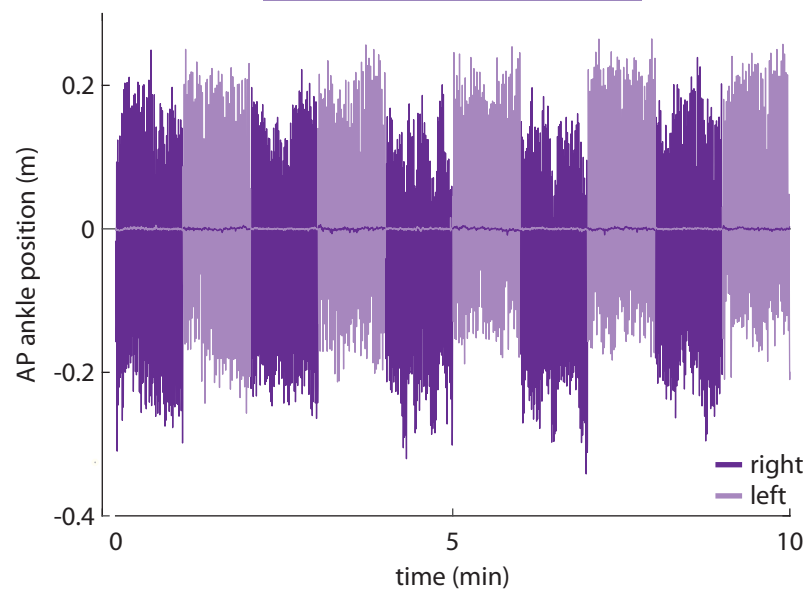

B

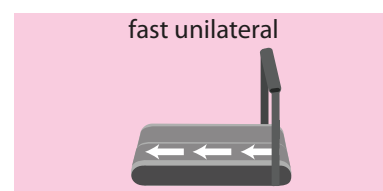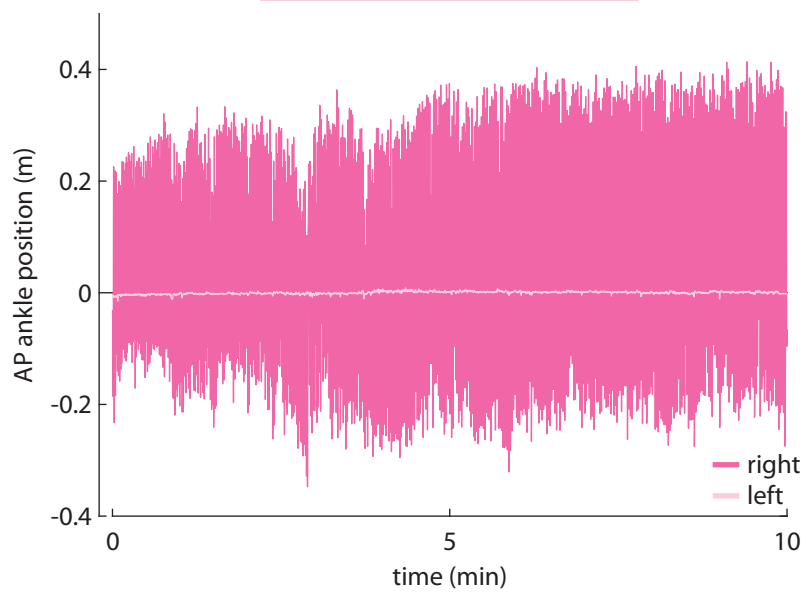

C

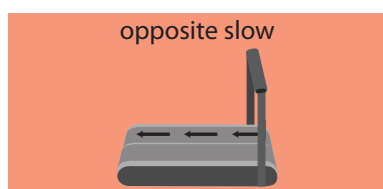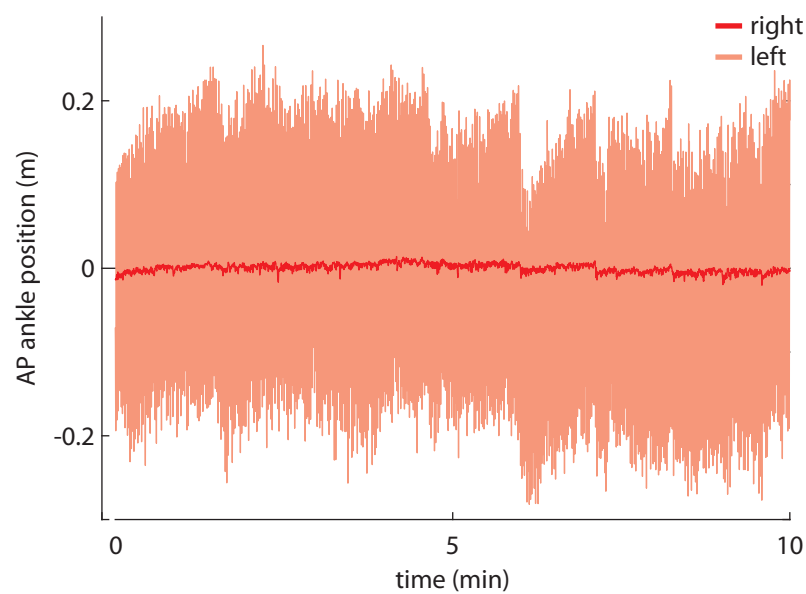

D

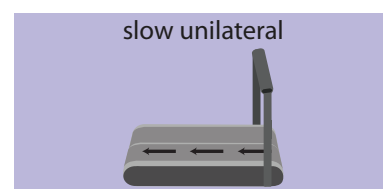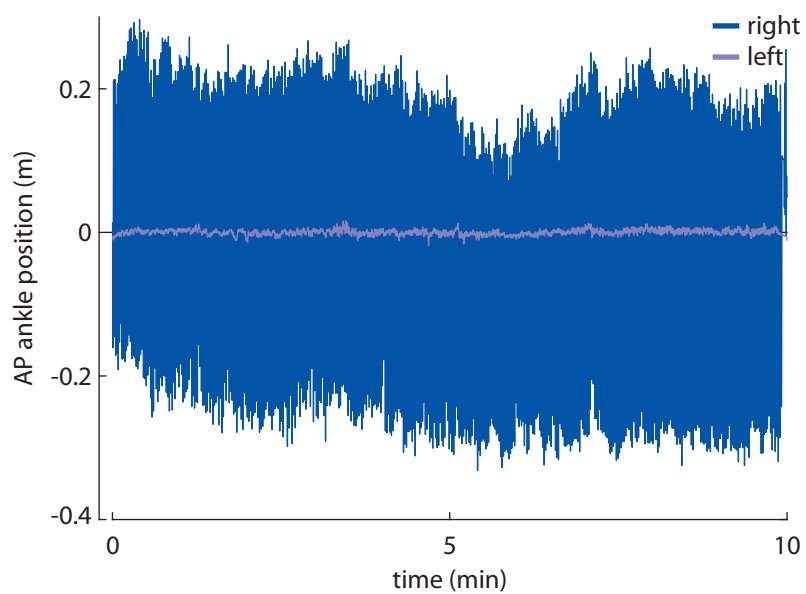

Supplement: Supplementary file 1 — Supplementary Figure 1. [file 41598_2020_75839_MOESM1_ESM.pdf]

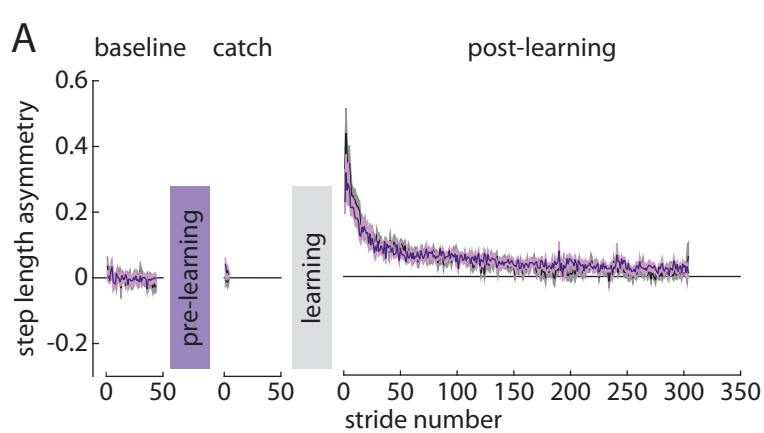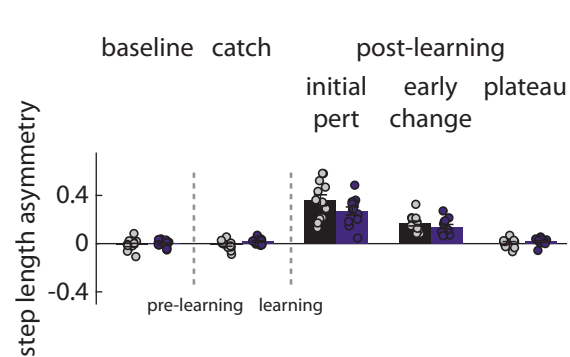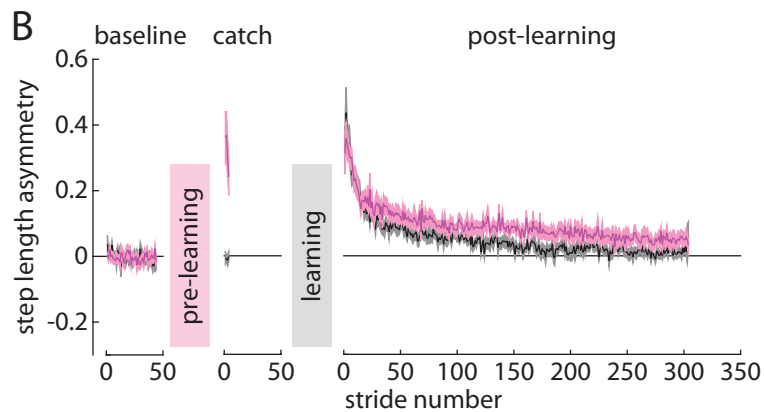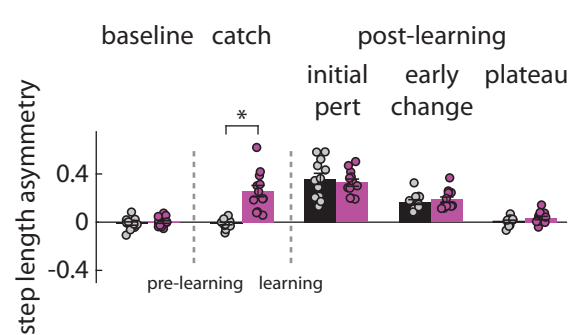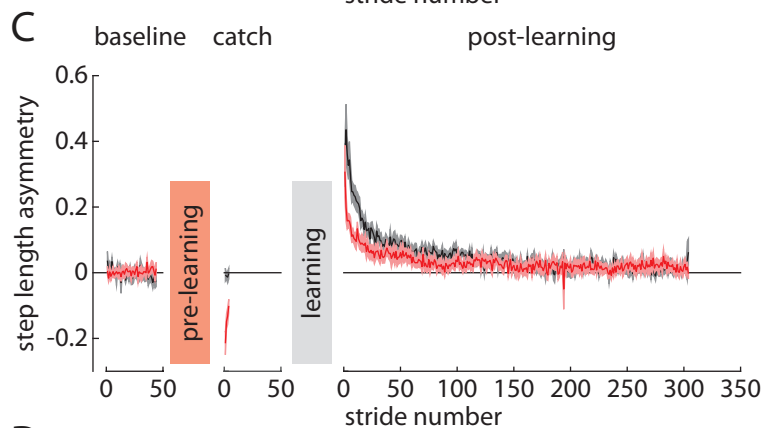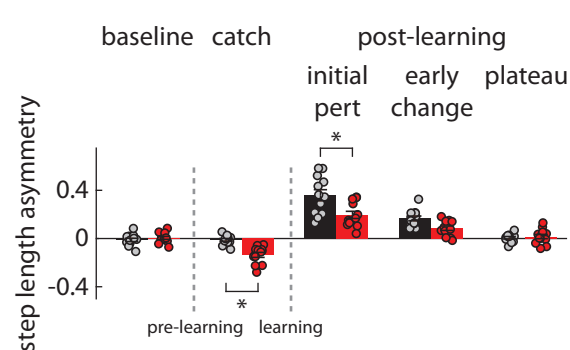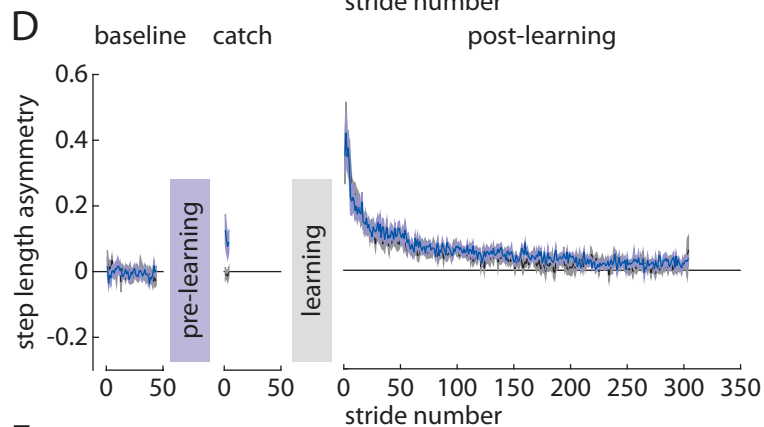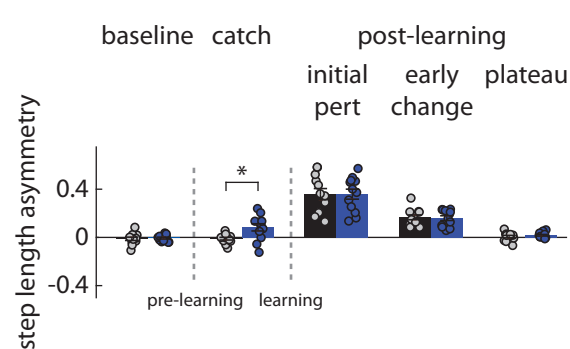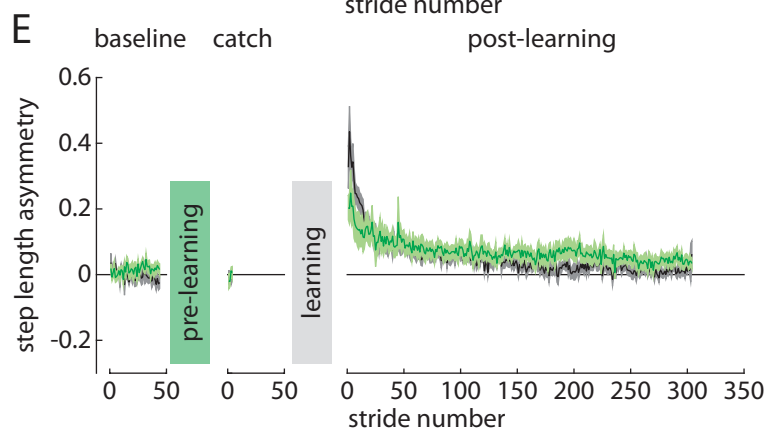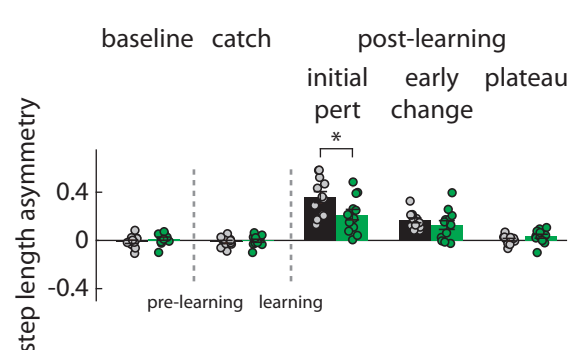

Supplement: Supplementary file 2 — Supplementary Figure 2. [file 41598_2020_75839_MOESM2_ESM.pdf]
