# Supplementary material for: Unilateral step training can drive faster learning of novel gait patterns
Source: Sci Rep. 2020 Oct 29;10:18628. doi: 10.1038/s41598-020-75839-3 (PMC7596053; doi:10.1038/s41598-020-75839-3)
Supplement: Supplementary file 3 — Supplementary Legends. [file 41598_2020_75839_MOESM3_ESM.docx]

Supplementary Material

**Unilateral step training can drive faster learning of novel gait patterns**

Christine N. Song^1,2^, Jan Stenum, PhD^1,3^, Kristan A. Leech, PhD, DPT, PT^4^, Chloe K. Keller^1,5^, and *Ryan T. Roemmich, PhD^1,3^

^1^Center for Movement Studies, Kennedy Krieger Institute, Baltimore, MD 21205

^2^Dept of Neuroscience, Johns Hopkins University School of Medicine, Baltimore, MD 21205

^3^Dept of Physical Medicine and Rehabilitation, Johns Hopkins University School of Medicine, Baltimore, MD 21205

^4^Division of Biokinesiology and Physical Therapy, University of Southern California, Los Angeles, CA 90033

^5^Fischell Department of Bioengineering, University of Maryland, College Park, MD 20742

*corresponding author

**FIGURE LEGENDS**

**Supplementary Figure 1**. Representative individual examples of foot movement (i.e., anterior-posterior ankle marker position) during the pre-learning conditions in the A) Alternating Unilateral, B) Fast Unilateral, C) Opposite Slow, and D) Slow Unilateral groups. Darker lines indicate right leg, lighter lines indicate left leg.

**Supplementary Figure 2**. Step length asymmetry during the baseline, catch, and post-learning periods in the A) Alternating Unilateral (purple), B) Fast Unilateral (pink), C) Opposite Slow (red), D) Slow Unilateral (blue), and E) Observational (green) groups compared to Control (black). * indicates p<0.05.
